# The Relationship Between Perceived Stress, State-Trait Anxiety, and Sleep Quality Among University Graduates in China During the COVID-19 Pandemic

**DOI:** 10.3389/fpsyg.2021.664780

**Published:** 2021-09-17

**Authors:** Bailong Liu, Ke Qiao, Youfeng Lu

**Affiliations:** School of Marxism, Xi’an University of Architecture and Technology, Xi’an, China

**Keywords:** sleep quality, anxiety, COVID-19, perceived stress, university graduates

## Abstract

The study aimed to investigate the relationship among perceived stress, state-trait anxiety, and sleep quality of graduates to provide a reference for improving their psychological status and attitude adjustment of job-searching during the COVID-19 pandemic. The research was conducted in a descriptive cross-sectional online survey between May 2020 and August 2020. The data were collected from 1,200 participants by using the personal information form prepared by the researchers in line with the literature, the Perceived Stress Scale, the State-Trait Anxiety Inventory, and the Pittsburgh Sleep Quality Index (PSQI). Among the surveyed participants, 47.67% were female, and 10.92% were medical students. The mean perceived stress, state anxiety, trait anxiety, and sleep quality were moderate and found as 31.4±6.69, 46.67±5.80, 49.45±5.54, and 5.94±2.47, respectively. The detection rates of state anxiety and trait anxiety were 48.63 and 49.50%, respectively. There was no significant difference in the detection rate of state anxiety and trait anxiety among different genders and majors (*p* >0.05). The detection rate of state anxiety and trait anxiety of rural family students was higher than that of urban family students (*p* <0.01). The score on the PSQI was positively associated with the scores on the perceived stress, state anxiety, and trait anxiety scales (*p* <0.001 for each model). Sleep quality was associated with increased perceived stress, state anxiety, and trait anxiety among graduates in China. Collectively, the study revealed the relationship between perceived stress, state-trait anxiety, and sleep quality among university graduates in China during the COVID-19 pandemic. Our results offer novel practical implications for all circles of the society to ensure students’ health under the context of the COVID-19 epidemic.

## Introduction

The outbreak of the COVID-19 emerged in Wuhan in December 2019 is an important public health problem (Hui et al., 2020; Li et al., 2020b). The WHO declared the outbreak of the novel coronavirus a global health emergency (World Health Organization, 2020). The COVID-19 outbreak is expected to continue in the coming years (Bao et al., 2020; Paules et al., 2020). It has been reported that the new infectious diseases may cause an increase in anxiety, depression, and stress in the general population (Erdoğan and Hocaoğlu, 2020; Tull et al., 2020; Kim et al., 2021). Facing the COVID-19 virus, a new infectious disease has resulted in a high prevalence of mental health problems in China and other countries (Chan et al., 2020; Choi et al., 2020; Ding et al., 2020). This increased stress in individuals, especially university students, can cause elevated anxiety levels and in turn compromise the sleep quality (Huang and Zhao, 2020; Zacher and Rudolph, 2020).

Meanwhile, the employment situation for university graduates is more and more serious. In recent years, the number of university graduates in China is gradually increasing, but the employment rate for university graduates is decreasing year by year (Li et al., 2020a). In 2020, the number of university graduates in China will reach 8.74 million. With the increasing uncertainty of social employment demand, the employment situation is complex and changeable. The employment of university graduates is facing severe challenges, and employment pressure is greatly increased (Liu et al., 2009; Hou et al., 2019).

It is extremely important to determine the factors that affect the health of university graduates in combating the COVID-19 pandemic. Anxiety is a normal reaction to the novel coronavirus pneumonia and employment pressure (Zhang et al., 2013; Gao et al., 2019). However, some students show overwhelming anxiety reactions, which would affect their daily function and employment mentality (Zhang et al., 2020). Sleep disorder is a common symptom of anxiety, which is one of the most concerned problems of university students and one of the main reasons for psychological counseling (Farrah et al., 2009). It is important for university graduates to obtain quality sleep to concentrate on completing their studies and actively look for jobs. Also, quality sleep plays an important role in defending against various infections (Ohrnberger et al., 2017; Besedovsky and Lange, 2019).

Under the dual influence of COVID-19 and the increasing employment pressure, the mental health and sleep disorders of university graduates have become increasingly prominent, which have attracted the attention of all sectors of society. In this study, 1,200 university graduates were selected to analyze their perceived stress, state anxiety, trait anxiety, and sleep quality to provide a reference for improving the mental health status of university graduates.

## Participants and Methods

### Participants and Sampling

The research was descriptive cross-sectional types. Only university graduates (aged≥18years) in Shaanxi Province who were able to provide informed consent were recruited in the study. A total of 1,200 university graduates from 11 universities in Shaanxi Province participated in the test. The survey time was from April to August 2020.

### Measurements

The data were collected by using the personal information form prepared by the researchers in line with the literature, the Perceived Stress Scale (PSS), the State-Trait Anxiety Inventory, and the PSQI. The study was carried out in the format of a “Questionnaire Star” electronic questionnaire system (Changsha Haoxing Information Technology Co., Ltd., China).

### Personal Information Form

This form contains the socio-demographic characteristics of university graduates at 11universities of Shaanxi Province, including age, gender, major, and origin.

#### Perceived Stress Scale

The PSS was developed by Cohen et al., which is used to assess the degree of stress an individual feel in the past month (Cohen et al., 1983). The Chinese version revised by Yang Tingzhong and Huang Hanteng was adopted (Yang and Huang, 2003). The scores of the PSS with 14 items that used vary between 0 and 56. The participants were evaluated of each item on a 5-point Likert scale ranging from “Never (0)” to “Very often (4).” Seven of the items containing positive statements were scored in reverse order. The high score indicated the excessive perception of stress (Eskin et al., 2013). The Alpha coefficient of the scale was found to be 0.86 (Yang and Huang, 2003).

#### State-Trait Anxiety Inventory

The State-Trait Anxiety Inventory is a test developed by Spielberger et al. that measures state and trait anxiety levels (Spielberger et al., 1970). The scale consists of two parts, the “state anxiety scale,” which is created with the aim of determining the instantaneous feelings, and the “trait anxiety scale,” which is created to determine the feelings in general (Yalcin et al., 2015; Kuroshm et al., 2021). Each test consists of 20, 4-point Likert-type questions. It is a four-degree scale ranging from “Nothing” to “All.” Scores from each form vary between 20 and 80, with higher scores indicating greater anxiety. A total score of S-AI>52 indicates state anxiety, and T-AI score>53 is defined as trait anxiety. The Chinese version of the inventory was revised by Ye Renmin in 1990 (Wang et al., 1999). It was stated that the State-Trait Anxiety Inventory (STAI) had an alpha value of 0.88 for reliability and 0.90 for validity (Wang et al., 1999).

#### Pittsburgh Sleep Quality Index

The PSQI is a questionnaire with 18 items assessing sleep quality over a 1-mo interval (Buysse et al., 1989; Okely et al., 2021). The scale was translated into Chinese by Liu Xianchen in 1996 (Liu et al., 1996). The 18 items are grouped into seven dimensions: subjective sleep quality, sleep latency, sleep disturbances, sleep duration, habitual sleep efficiency, use of sleeping medication, and daytime dysfunction. The sum of scores for these seven dimensions yields a composite score, ranging from 0 to 21 (Xiao et al., 2020; Jacopo et al., 2021). Higher scores indicate worse sleep quality. A total score of PSQI≥8 indicates poor sleep quality, 5~7 indicates average sleep quality, and ≤4 indicates good sleep quality. The factor score of PSQI≥2 indicates poor or very poor sleep quality on this factor. The reliability and validity of the scale were 0.99 and 0.85, respectively (Liu et al., 1996).

### Statistical Methods

In the data collection process, measurement tools were applied by applying an online data collection method. This work lasted 4months, using “Personal Information Form,” “PSS,” “State-Trait Anxiety Inventory,” and “PSQI.”

The data were analyzed using the SPSS version 23.0 software package. Percentages, Kruskal-Wallis, *t*-test, chi-square test, and correlations tests were used to evaluate the data. Kappa and correlation analyses were conducted to determine consistency among the observers. A value of *p*<0.05 was considered statistically significant.

## Results

### Descriptive Results

The socio-demographic characteristics of a total of 1,200 participants were showed in Table 1. Among them, there were 628 males and 572 females. The average age was 23.36±2.72years, from 21 to 26. A total of 624 students (52%) majored in science and engineering, 445 students (37.08%) majored in liberal arts, and 131 students (10.92%) majored in medicine. A total of 52.42% of the participants came from urban families, while 47.58% of them were rural students.

**Table 1:** Socio-demographic characteristics of the study sample.

Characteristics		*n*(%)
Gender	Male	628(52.33)
	Female	572(47.67)
Major	Science and Engineering	624(52.0)
	Liberal Arts	445(37.08)
	Medicine	131(10.92)
Origin	Urban	629(52.42)
	Rural	571(47.58)
Age(Mean±SD)		23.36±2.72

The mean scores of the sample on the PSS, STAI, and PSQI were showed in Table 2. The mean perceived stress, state anxiety, trait anxiety, and sleep quality were moderate and found as 31.4±6.69, 46.67±5.80, 49.45±5.54, and 5.94±2.47, respectively.

**Table 2:** Mean scores of the sample on the PSS, STAI, and PSQI.

	M + N-MAX	Mean(SD)
Perceived Stress Scale	0~56	31.44 ± 6.69
State Anxiety	20~80	46.67 ± 7.80
Trait Anxiety	20~80	49.25 ± 7.54
Sleep Quality Scale	0~21	5.94 ± 2.47

### Correlations Between Outcomes

The average scores of state anxiety and trait anxiety were 46.67±7.80 and 49.25±7.54, respectively. The detection rates of state anxiety and trait anxiety among 1,200 university graduates were 48.42 and 49.25%, respectively. As shown in Table 3, there was no significant difference in the detection rates of state anxiety and trait anxiety among university graduates of different genders and majors (*p*≥0.05). The detection rates of state anxiety and trait anxiety in rural students were higher than those in urban areas, and the differences were statistically significant (*p*<0.01).

**Table 3:** The comparison of socio-demographic characteristics between the detection rate of state anxiety and trait anxiety.

Characteristics		n	Test, *p* value	State Anxiety (Detection rate/%)	Trait Anxiety (Detection rate/%)
Gender	Male	628		294(46.82%)	299(47.61%)
	Female	572		287(50.17%)	292(51.05%)
			*χ* ^2^	0.89	1.00
			p	>0.05	>0.05
Major	Science and Engineering	624		297(47.60%)	303(48.56%)
	Liberal arts	445		219(49.21%)	226(50.79%)
	Medicine	131		65(49.62%)	62(47.33%)
			*χ* ^2^	0.18	0.07
			p	>0.05	>0.05
Origin	Urban	629		243(38.63%)	252(40.06%)
	Rural	571		338(59.19%)	339(59.37)
			*χ* ^2^	31.39	22.29
			p	<0.01	<0.01

The total average score of PSQI was 5.94±2.47. As shown in Table 4, according to the evaluation standard, among 1,200 university graduates, 27.83% had poor sleep quality, 54.83% had average sleep quality, and 17.33% had good sleep quality. The main symptoms of poor sleep were 77.92% daytime dysfunction, 47.08% sleep duration, 27.33% subjective sleep quality, and 24.25% sleep latency. There were significant differences in the distribution of sleep quality among university graduates from different majors and origins (*p*<0.01).

**Table 4:** The comparison of socio-demographic characteristics in the distribution of sleep quality.

Characteristics		*n*	Poor sleep quality (Constituent Ratio/%)	Average sleep quality (Constituent Ratio/%)	Good sleep quality (Constituent Ratio/%)	*χ* ^2^	*p*
Gender	Male	628	173(27.55)	344(54.78)	111(17.68)	3.04	>0.05
	Female	572	161(28.15)	314(54.90)	97(16.96)		
Major	Science and Engineering	624	158(25.32)	359(57.53)	107(17.15)	13.54	<0.01
	Liberal arts	445	139(31.24)	230(51.69)	76(17.08)		
	Medicine	131	37(28.24)	69(52.67)	25(19.08)		
Origin	Urban	629	153(24.32)	328(52.15)	148(23.53)	16.33	<0.01
	Rural	571	181(31.70)	330(57.79)	60(10.51)		

The relationships between the socio-demographic characteristics and mean scores of the sample on the PSS, STAI, and PSQI were showed in Table 5. According to the results, there was no statistically significant correlation between perceived stress scores and socio-demographic characteristics. State anxiety levels were significantly higher in woman students than man students (*p*<0.05). University graduates who came from rural families had significantly higher state anxiety levels (*p*<0.05). There were no significant differences found in terms of trait anxiety levels.

**Table 5:** The comparison of socio-demographic characteristics between mean scores of the sample on the PSS, STAI, and PSQI.

Characteristics		PSS score(M±SD)	S-AI score(M±SD)	T-AI score(M±SD)	PSQI score(M±SD)
Gender	Male	30.88±6.47	45.49±7.04	48.59±8.12	5.82±2.51
	Female	32.05±6.93	47.96±8.63	49.98±6.91	6.07±2.43
	Test, p value	*t* =−1.324	*t* =2.803	*t* =−1.951	*t* =1.477
		*p* =0.184	*p* =0.005	*p* =0.053	*p* =0.141
Major	Science and Engineering	30.87±6.31	46.94±9.09	49.81±8.24	5.82±2.41
	Liberal arts	32.24±5.98	46.63±8.52	48.86±6.79	6.11±2.49
	Medicine	31.41±7.33	45.67±6.92	47.92±6.75	5.93±2.68
	Test, p value	KW=4.077 *p* =0.231	KW=1.124 *p* =0.737	KW=0.902 *p* =0.813	KW=0.902 *p* =0.813
Origin	Urban	30.82±7.09	45.73±7.34	49.13±7.03	5.69±2.62
	Rural	31.94±6.24	47.68±8.27	49.36±8.12	6.23±2.31
	Test, p value	*t* =0.356 *p* =0.722	*t* =1.967 *p* =0.050	*t* =0.136 *p* =0.892	*t* =1.113 *p* =0.127

According to the correlation analysis in Table 6, there was no significant relationship between state anxiety and perceived stress (*r*=−0.037, *p*=0.611). There was also no significant relationship between sleep quality and perceived stress (*r*=−0.037, *p*=0.611). However, a positive relationship was found between the trait anxiety and perceived stress (*r*=0.164, *p*=0.016), state anxiety and trait anxiety (*r* =0.520, *p*=0.000), state anxiety and sleep quality (*r*=0.157, *p*=0.021), and trait anxiety and sleep quality (*r*=0.142, *p*=0.041).

**Table 6:** Correlations between the PSS, the STAI, and the PSQI.

Characteristics		PSS	S-AI	T-AI	PSQI
PSS	r		−0.037	0.164	−0.037
	P		0.611	0.016	0.607
S-AI	r	−0.037		0.520	0.157
	P	0.611		0.000	0.021
T-AI	r	0.164	0.520		0.142
	P	0.016	0.000		0.041
PSQI	r	−0.037	0.157	0.142	
	P	0.607	0.021	0.041	

### Predictors of Change

Taking state anxiety and trait anxiety as dependent variables, sleep quality score as independent variables, and gender, major category, and origin as control variables, binary logistic regression analysis was conducted. The results in Table 7 showed that the evaluation results and total scores of sleep quality of university graduates were positively correlated with state anxiety and trait anxiety.

**Table 7:** Regression analysis on the relationship between sleep quality and state-trait anxiety [n=1,200 odd ratio (OR, 95%CI)].

Sleep quality	State anxiety	Trait anxiety
Subjective sleep quality	2.02 (1.73~2.94)^**^	2.07 (1.49~2.16)^**^
Sleep duration	1.81 (1.71~1.99)^**^	1.98 (1.68~3.92)^**^
Sleep latency	2.84 (1.65~3.12)^*^	3.08 (1.47~3.91)^**^
Daytime dysfunction	3.24 (1.86~3.68)^*^	3.37 (2.67~4.81)^*^
Habitual sleep efficiency	2.07 (1.65~2.57)^*^	2.21 (1.68~3.95)^*^
Use of sleeping medication	3.20 (1.98~4.34)^*^	3.64 (1.92~3.80)^*^
Sleep disturbances	2.98 (1.33~3.09)^**^	3.11 (1.72~4.00)^*^
Total score	2.57 (1.86~2.97)^**^	3.04(2.04~3.71)^*^

* *p<0.05*

*and*

** *p<0.01*.

Taking trait anxiety as the independent variable and the total score of PSQI and its factors (subjective sleep quality and sleep duration) as the dependent variable, the regression equations were constructed. The results were showed in Table 8. Trait anxiety had a significant predictive effect on subjective sleep quality, sleep duration, and PSQI total score, and the explained amount of variation was 2.56, 2.69, and 1.84%, respectively.

**Table 8:** Regression analysis of trait anxiety and sleep quality.

	Δ*R*^2^	*F*	*β*	*t*
Subjective sleep quality	0.03	12.37	0.16	3.51^**^
Sleep duration	0.01	5.44	0.11	2.33^*^
PSQI total score	0.02	11.58	0.16	3.39^**^

* *p<0.05*

*and*

** *p<0.01*.

## Discussion

In the fight against the COVID-19 pandemic, it is extremely important to identify the factors that affect the psychological health of university graduates. A general picture of the psychological state of university graduates in China during the COVID-19 pandemic has been presented. Pandemics have many negative effects on society, economy, psychology, and spirit (Fong et al., 2020; Gao et al., 2020; Salari et al., 2020; Wang et al., 2020; Zhang and Ma, 2020; Li et al., 2020c). Under the pressure of study and employment, the mental health of university graduates has always been the focus of colleges and society (Kahn, 2010; Altonji et al., 2016). Therefore, the impact of novel coronavirus pneumonia, the fear of leaving campus, fierce competition, huge employment pressure, and worries about future development can cause serious mental health problems for university graduates. Lack of sleep or poor sleep quality can lead to college students’ fatigue, inattentiveness, low learning efficiency, and other undesirable phenomena (Gadie et al., 2017; Barros et al., 2019; Cao et al., 2021).

Through this cross-sectional study, the mental health problems and the associated factors among Chinese university graduates with pressure increases exposed to COVID-19 were assessed. The degrees of the perceived stress, anxiety, and sleep quality of 1,200 participants were assessed using the Perceived Stress Scale, the State-Trait Anxiety Inventory, and the PSQI, respectively, which found that the mean perceived stress, state anxiety, trait anxiety, and sleep quality were moderate and found as 31.4±6.69, 46.67±5.80, 49.45±5.54, and5.94±2.47, respectively.

The findings of this study demonstrated that the perceived stress, anxiety, and sleep quality of university graduates were affected by some demographic variables. The results showed that the detection rates of state anxiety and trait anxiety were 48.63 and 49.50%. A considerable number of university graduates had different degrees of state anxiety and trait anxiety, which was significantly higher than the detection rates of state anxiety and trait anxiety on the adult population in other studies (Madrid-Valero et al., 2017). The reason for this analysis is that the university graduates will face more pressure, unclear future planning, and fierce competition for employment under the influence of COVID-19. This shows that during the period of COVID-19, universities and all sectors of society should give university graduates more care and psychological support to help them to clear up negative emotions and avoid serious harm to their mental health.

In addition, the detection rates of state anxiety and trait anxiety of different genders and different majors were similar, which indicated that there was a high risk of anxiety for both males and females, regardless of their majors. This study found that state anxiety levels were significantly higher in females than males (*p*<0.05). This was consistent with the greater pressure on female employment in the current society. The detection rates of state anxiety and trait anxiety on rural students were higher than those in urban areas. It was speculated that it may be due to the low financial support of rural families for further study and the great economic pressure of employment for their families. Serving as reserve talents for the healthcare system, medical students were not yet professionally matured enough to face one of the worst global public health crises. The perceived stress and anxiety induced by the COVID-19 epidemic might affect medical students’ future career choice (Zheng et al., 2021).

There was a close relationship between state-trait anxiety and sleep quality of university graduates. Multivariable logistic regression analysis was performed to identify factors associated with mental health outcomes among university graduates during COVID-19. The regression analysis showed that each factor and total score of sleep quality were positively correlated with state and trait anxiety. They can influence each other and form a vicious circle (Liu et al., 2016). The relationship between sleep quality and anxiety should be paid attention in order to reduce the state and trait anxiety of university graduates. The results of the study showed that trait anxiety had a significant predictive effect on subjective sleep quality, sleep duration, and PSQI total score.

## Conclusion

The study managed to capture some immediate positive and negative mental health impacts of the COVID-19 pandemic. The results showed that rural graduates looking for employment, especially students majored in liberal arts, were found to have a high risk of mental health symptoms that were not conducive to development and may need psychological support or interventions. Since the COVID-19 pandemic is still ongoing, these findings need to be confirmed and investigated in future.

## Limitations

This study is a cross-sectional study, which can not reveal the causal direction of the relationship between perceived stress, state-trait anxiety, and sleep quality. It needs to carry out a prospective cohort study to explore.

The effect of state anxiety on sleep quality is direct and significant. The trait anxiety can trigger and cause all kinds of sleep problems by influencing individual anxiety tendency steadily. In this study, the explanation rate of trait anxiety to the variance of PSQI is not high, which may also be due to this. Trait anxiety has an indirect effect on sleep quality, which mainly affects sleep quality through the mediating effect of state anxiety.

## Data Availability Statement

The raw data supporting the conclusions of this article will be made available by the authors, without undue reservation.

## Ethics Statement

The studies involving human participants were reviewed and approved by the Ethics Committee of Xi’an University of Architecture and Technology. The patients/participants provided their written informed consent to participate in this study.

## Author Contributions

BL conceived the initial idea for the study and helped to study design. KQ designed the study, collected the data, performed the statistical analysis, and helped to recruit the participants. BL and YL contributed to intervention design and draft the manuscript. All authors have read and agreed to the published version of the manuscript.

## Funding

This study is supported by the Excellent Project of University Student Work in Shaanxi Province (2020FXM06).

## Conflict of Interest

The authors declare that the research was conducted in the absence of any commercial or financial relationships that could be construed as a potential conflict of interest.

## Publisher’s Note

All claims expressed in this article are solely those of the authors and do not necessarily represent those of their affiliated organizations, or those of the publisher, the editors and the reviewers. Any product that may be evaluated in this article, or claim that may be made by its manufacturer, is not guaranteed or endorsed by the publisher.
